# Solving the Puzzle: A Compelling Case Study of Calcaneal Apophysitis With Achilles Tendon Calcification in a 14-Year-Old Patient

**DOI:** 10.7759/cureus.66677

**Published:** 2024-08-12

**Authors:** Souhail Yachaoui, Berrahal Lokman, Araj Aymane, Houssam Mahla, Ahmed Amine El Oumri

**Affiliations:** 1 Physical Medicine and Rehabilitation, Faculty of Medicine and Pharmacy, Mohammed VI University Hospital, Mohamed I University, Oujda, MAR

**Keywords:** diagnosis methods, growth plate stress, youth sport injuries, shockwave treatment, foot and ankle disorders, heel pain

## Abstract

Calcaneal apophysitis, known as Sever's disease, manifests as heel pain and is prevalent among children and adolescents, particularly during growth spurts and periods of heightened physical activity. Although Sever's disease is well-documented, its co-occurrence with other foot pathologies in pediatric patients is relatively uncommon.

We present here a unique case of a 14-year-old female patient who presented with significant heel pain and discomfort associated with flat feet, impacting her daily activities and physical performance. Clinical examination revealed tenderness at the heel consistent with Sever's disease, along with symptoms suggestive of posterior tibial tendinopathy and radiographic evidence of Achilles tendon calcification. The primary diagnoses included Sever's disease, posterior tibial tendinopathy, and calcification of the Achilles tendon. Management involved a thorough assessment comprising physical examination and imaging studies to confirm the diagnoses. Pharmacological and non-pharmacological interventions such as activity modification, stretching exercises, and orthotic devices were implemented to alleviate symptoms and improve foot mechanics. Over the course of treatment, the patient showed gradual improvement in pain levels and functional abilities, indicating a positive response to therapy. Long-term follow-up aimed at preventing recurrence and optimizing foot health was recommended to ensure sustained recovery and overall well-being.

In this case study, we aim to elucidate the clinical presentation, diagnostic challenges, and management approach employed in addressing these concurrent foot conditions. By exploring this case, we hope to contribute valuable insights to the understanding and management of pediatric foot pathologies, particularly in cases involving multiple co-existing conditions.

## Introduction

Calcaneal apophysitis, also known as Sever's disease, is a common source of heel pain among physically active children aged eight to 15 years, akin to Osgood-Schlatter disease (OSD). While OSD typically affects the distal patellar tendon, calcaneal apophysitis primarily involves the growth plate of the calcaneus and may include inflammation of the Achilles tendon [1,2]. Despite its significance, it often goes undiagnosed by emergency department providers [3].

The condition arises from repetitive stress and overuse, leading to microtrauma at the calcaneal growth plate [4]. Risk factors include high levels of physical activity and obesity. Diagnosis hinges on clinical assessment, notably heel tenderness upon palpation and compression. Radiographs can aid in diagnosis, but ultrasound is crucial for confirming inflammation and severity [1].

Magnetic resonance imaging plays a pivotal role in evaluating the extent of inflammation, providing critical insights for accurate diagnosis and management. Comparing calcaneal apophysitis with OSD enhances diagnostic and treatment strategies in pediatric orthopedics [1-5].

This case report aims to explore calcaneal apophysitis by presenting the case of a 14-year-old patient with a 10-month history of heel pain, highlighting clinical features and diagnostic considerations.

## Case presentation

A 14-year-old female with no significant pathological familial history, no current medication use, and no previous trauma, BMI 22 kg/m^2^, presented to our department with complaints of persistent pain in the right heel, particularly exacerbated during weight-bearing activities such as walking and running. The patient, an active participant in school sports, reported a gradual onset of symptoms over the past few months without any history of trauma or significant medical illness. She described the pain as localized to the heel with occasional radiation along the posterior aspect of the ankles. Notably, she mentioned walking approximately 10 kilometers per day to commute to and from school. There were no associated systemic symptoms such as fever, night sweats, or weight loss.

Upon examination, the patient demonstrated bilateral pes planus with reduced medial longitudinal arch height and mild hindfoot valgus. Tenderness was noted over the posterior aspect of both heels, particularly at the insertion of the Achilles tendon. Palpation along the course of the posterior tibial tendon elicited discomfort. Active and passive dorsiflexion of the ankles exacerbated the pain. Additionally, a noticeable valgus deformity of the feet was observed. It is noteworthy that we performed weight-bearing radiographs of the feet, revealing bilateral flattening of the calcaneal apophyses with mild irregularity and fragmentation, consistent with calcaneal apophysitis.

Furthermore, the patient underwent a podoscope examination to assess the weight distribution and foot morphology. The podoscope confirmed the presence of flat feet and highlighted the noticeable valgus alignment. This objective assessment corroborated our clinical findings and provided valuable insight into the biomechanical factors contributing to the patient's symptoms.

**Figure 1:**
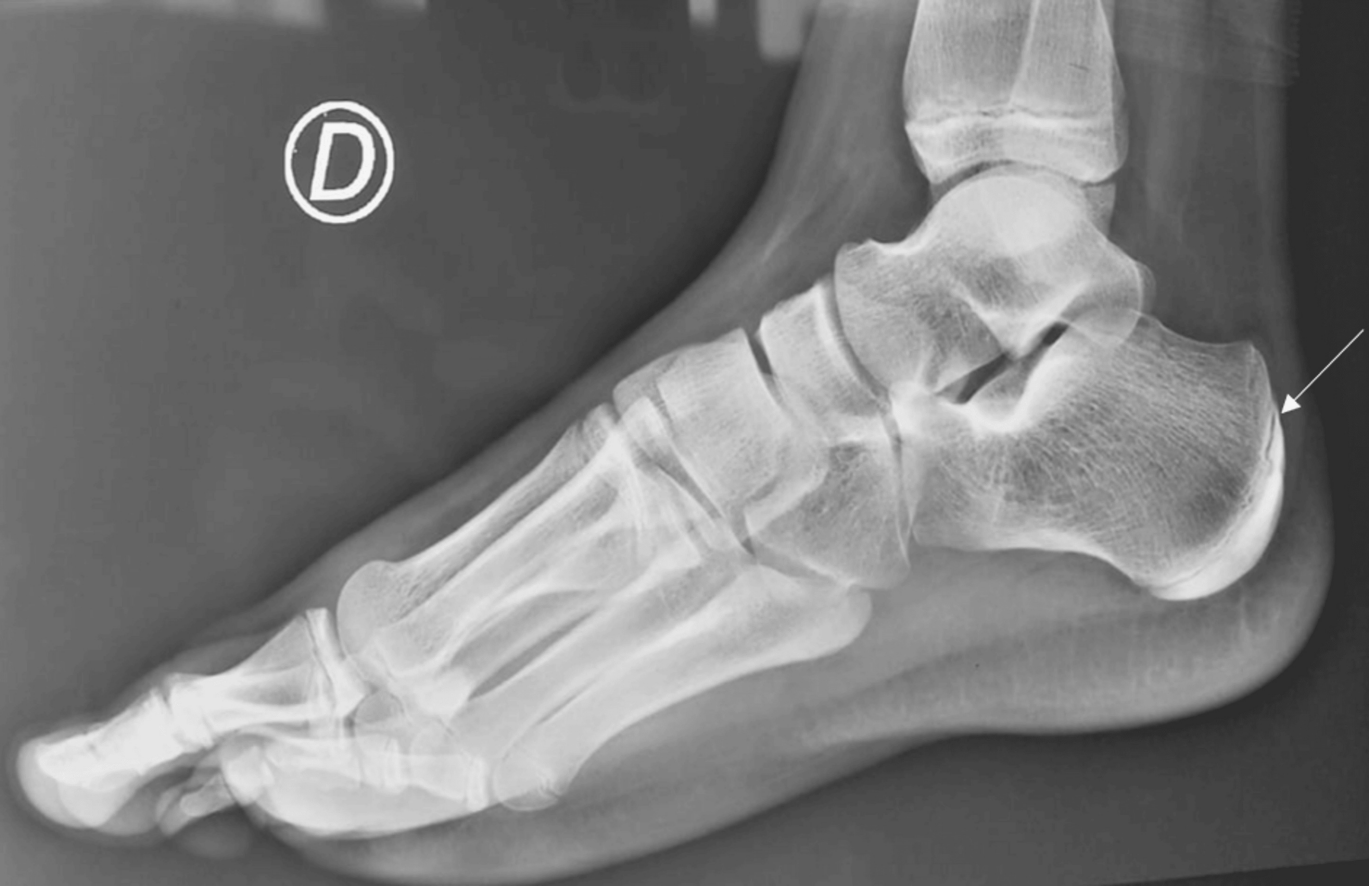
Lateral radiograph of the right (D) foot demonstrating the presence of calcaneal apophyseal fragmentation (white arrow)

Notably, the radiographs also demonstrated the angles of Djian-Annonier measuring 137 degrees, indicating significant valgus alignment and the presence of calcaneal apophyseal fragmentation (Figure 1).

Routine laboratory investigations, including complete blood count (CBC), erythrocyte sedimentation rate (ESR), and C-reactive protein (CRP) levels, were within normal limits. Additionally, electrolyte levels were unremarkable. These findings supported the absence of systemic inflammation or underlying metabolic abnormalities contributing to the patient's symptoms.

**Figure 2:**
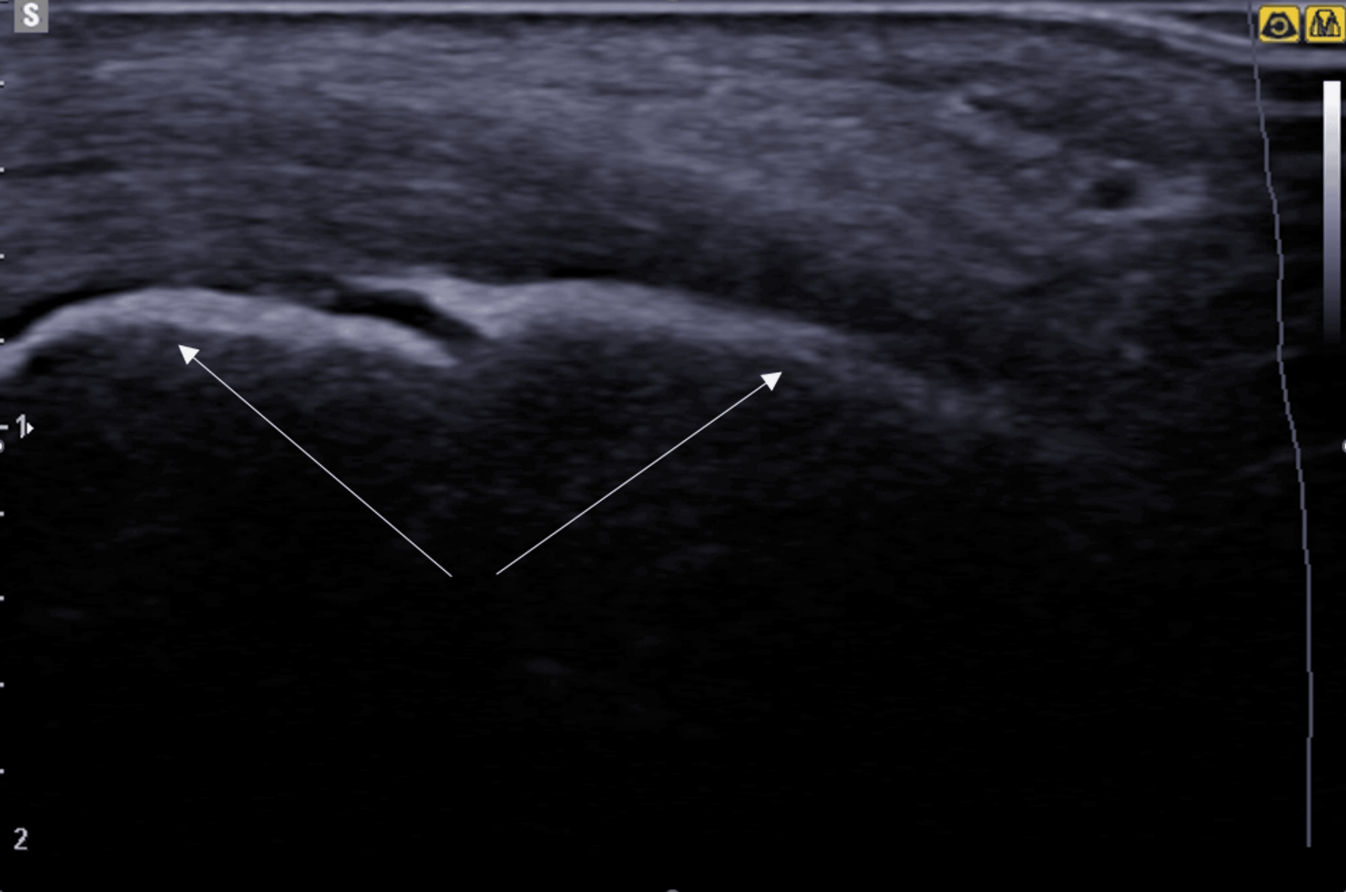
Ultrasound of the right calcaneus (white arrows) longitudinal section showing the presence of fragmentation of the calcaneal apophysis consistent with calcaneal apophysitis

**Figure 3:**
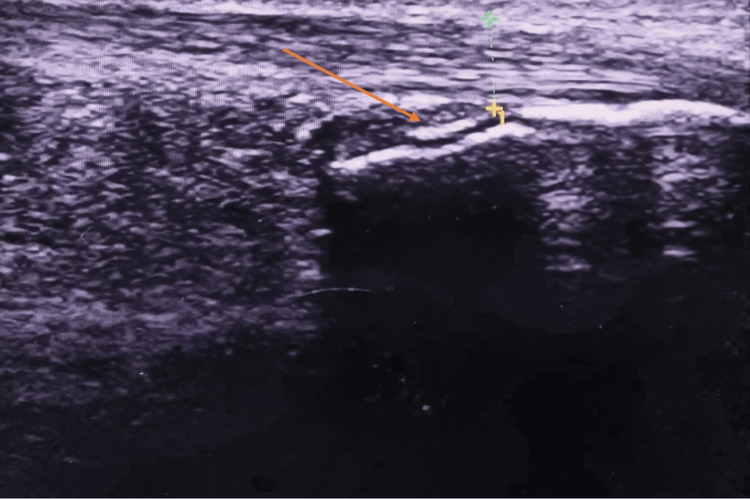
Ultrasound of the fragmented calcaneus longitudinal section showing the calcification at the right Achilles tendon (orange arrow)

During the ultrasound examination of both feet, a fragmentation of the right calcaneus (Figure 2) was observed with calcification at the right Achilles tendon (Figure 3), also an effusion in the posterior tibial tendon, while the Doppler examination was negative and showed no hyperemia.

The imaging results were significant in showing the absence of retrocalcaneal bursitis, plantar fasciitis, or thickening of the Achilles tendon.

During the diagnostic evaluation of the patient presenting with persistent heel pain and related symptoms, several differential diagnoses were considered and ruled out based on clinical findings and investigative procedures. These included the following:

Retrocalcaneal bursitis: Evaluated due to localized heel tenderness, but imaging studies did not indicate inflammation or swelling around the bursa, ruling out this condition.

Tarsal coalition: Considered due to the patient's pes planus and hindfoot valgus deformity, but ruled out based on imaging findings that primarily showed calcaneal apophysitis without fusion abnormalities.

Ankle sprain or fracture: Excluded based on the absence of trauma history and radiographic evidence ruling out acute bone injuries.

Rheumatologic conditions: Considered due to persistent pain and involvement of multiple foot structures, but laboratory investigations (CBC, ESR, CRP) were within normal limits, ruling out systemic inflammatory conditions.

By detailing these considerations, the differential diagnosis process provides a comprehensive understanding of the diagnostic pathway pursued to arrive at the primary diagnoses of Sever's disease and calcification of the Achilles tendon.

**Figure 4:**
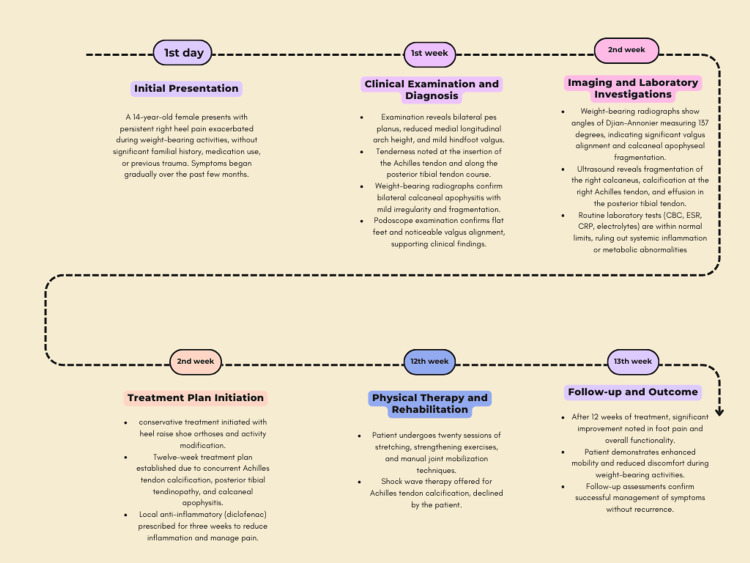
Timeline of events in the episode of care

The patient was counseled to utilize heel raise shoe orthoses and to refrain from participating in sports activities that could worsen the condition. Due to the concurrent presence of Achilles tendon calcification and the posterior tibial tendinopathy and calcaneal apophysitis, an extended treatment duration of 12 weeks was established (Figure 4).

A local anti-inflammatory, diclofenac, was prescribed for three weeks with the aim of reducing inflammation and providing analgesic relief.

The patient received stretching and strengthening exercises, including a regimen of 20 sessions of manual stretching along with joint mobilization techniques. Despite being offered shock wave therapy sessions for the calcification of the Achilles tendon, the patient chose not to pursue this treatment option.

After 12 weeks of rehabilitation and treatment, including conservative management strategies and physical therapy, the patient showed notable improvement, with a significant reduction in foot pain and enhanced overall functionality.

This timeline (Figure 4) organizes historical and current information from the episode of care, illustrating the chronological sequence of events.

## Discussion

The key result of this study was effectively addressing calcaneal apophysitis in the case presented, using a combination of approaches; attempting to identify the associated signs, in our study, we found calcification of the Achilles tendon and effusion of the posterior tibial tendon [4].

The primary hypothesis for the pathogenesis of calcaneal apophysitis injury is mechanical overuse, which results from repetitive impact pressure and shear stresses on the open growth plate of the calcaneus. Elevated levels of physical activity and obesity are identified as the predominant risk factors contributing to the development of calcaneal apophysitis in pediatric patients [1-6]. Additionally, a research study found a significant association between calcaneal apophysitis and higher BMI, increased weight, and greater height among affected individuals [7]. 

In the context of this specific case, these general principles align with the patient’s presentation and support the conclusions drawn. The 14-year-old female in this case was an active participant in school sports, walking approximately 10 kilometers daily to commute to and from school. This high level of physical activity corresponds with the mechanical overuse hypothesis, as repetitive stress and impact likely contributed to her heel pain. Although her BMI was 22, which is within the normal range and not indicative of obesity, her significant physical activity level and the mechanical stress from extensive walking align with the proposed pathogenesis of calcaneal apophysitis.

The absence of significant systemic symptoms and trauma, combined with the gradual onset of localized heel pain exacerbated by weight-bearing activities, further supports the diagnosis of calcaneal apophysitis. The impact of high activity levels and the absence of obesity as a factor highlight that while elevated BMI is a known risk factor, it is not the sole contributor to calcaneal apophysitis. Instead, the mechanical stresses from her daily activities played a significant role in her condition.

During the physical examination, we notice tenderness upon palpation and compression at the medial and lateral aspects of the heel. Usually, there is no redness or swelling. Limited ankle dorsiflexion is common, and physical activity tends to worsen the pain [1-4]. The diagnosis can be confirmed with a "squeeze test," where applying pressure to the heel causes pain [8].

Radiographic findings in calcaneal apophysitis injury often show increased density and fragmentation of the calcaneal apophysis, aiding diagnosis but not exclusively indicative [5]. Plain films are vital to rule out other causes like fractures or bone cysts. They ensure an accurate diagnosis by excluding alternative etiologies [9].

Ultrasound detects pathologic findings like pretibial swelling, ossification center fragmentation, patellar tendon thickening, and fluid collection in the infrapatellar bursa, making it a reliable diagnostic tool for calcaneal apophysitis-related changes [1-10]. In most cases, radiological exams find no Achilles tendon abnormalities when using ultrasound to assess the calcaneal apophysis [11].

The treatment of calcaneal apophysitis employs a multifaceted approach involving several modalities that have been extensively researched for their effectiveness. Pain in calcaneal apophysitis is closely tied to physical activity, so it is recommended to modify or restrict activities during painful episodes. Treatment strategies include ice therapy, non-steroidal anti-inflammatory drugs for pain relief, and stretching exercises aimed at calf muscles to alleviate stress on the Achilles tendon. Additionally, heel cups and taping techniques are utilized to provide support and stability to the affected area [1].

Shock wave therapy has demonstrated effectiveness in addressing tendon calcifications associated with calcaneal apophysitis [12]. In more severe cases or when patients are noncompliant with conservative measures, immobilization using a boot or cast may be necessary. This approach is often combined with rehabilitation therapies to optimize treatment outcomes [6].

## Conclusions

In conclusion, this case highlights the successful management of calcaneal apophysitis in a 14-year-old through a multimodal approach. Accurate diagnosis, achieved through clinical examination and ultrasound that revealed characteristic ossification center fragmentation, was key. The patient's significant improvement within 12 weeks underscores the value of early intervention and a comprehensive treatment plan for optimal outcomes in calcaneal apophysitis.
